# Could SGLT2 Inhibitors Improve Exercise Intolerance in Chronic Heart Failure?

**DOI:** 10.3390/ijms23158631

**Published:** 2022-08-03

**Authors:** Suzanne N. Voorrips, Huitzilihuitl Saucedo-Orozco, Pablo I. Sánchez-Aguilera, Rudolf A. De Boer, Peter Van der Meer, B. Daan Westenbrink

**Affiliations:** Department of Cardiology, University Medical Center Groningen, University of Groningen, 9713 GZ Groningen, The Netherlands; h.saucedo.orozco@umcg.nl (H.S.-O.); p.i.sanchez.aguilera@umcg.nl (P.I.S.-A.); r.a.de.boer@umcg.nl (R.A.D.B.); p.van.der.meer@umcg.nl (P.V.d.M.)

**Keywords:** SGLT2 inhibitors, heart failure, exercise intolerance, exercise capacity, mitochondria, metabolism, skeletal muscle, cardiac effects, cardiac function

## Abstract

Despite the constant improvement of therapeutical options, heart failure (HF) remains associated with high mortality and morbidity. While new developments in guideline-recommended therapies can prolong survival and postpone HF hospitalizations, impaired exercise capacity remains one of the most debilitating symptoms of HF. Exercise intolerance in HF is multifactorial in origin, as the underlying cardiovascular pathology and reactive changes in skeletal muscle composition and metabolism both contribute. Recently, sodium-related glucose transporter 2 (SGLT2) inhibitors were found to improve cardiovascular outcomes significantly. Whilst much effort has been devoted to untangling the mechanisms responsible for these cardiovascular benefits of SGLT2 inhibitors, little is known about the effect of SGLT2 inhibitors on exercise performance in HF. This review provides an overview of the pathophysiological mechanisms that are responsible for exercise intolerance in HF, elaborates on the potential SGLT2-inhibitor-mediated effects on these phenomena, and provides an up-to-date overview of existing studies on the effect of SGLT2 inhibitors on clinical outcome parameters that are relevant to the assessment of exercise capacity. Finally, current gaps in the evidence and potential future perspectives on the effects of SGLT2 inhibitors on exercise intolerance in chronic HF are discussed.

## 1. Introduction

### 1.1. Current Developments in the Treatment of Heart Failure

Heart failure (HF) is a clinical syndrome that is characterized by severe symptoms of dyspnea, fatigue, and exercise intolerance [1,2,3]. With an estimated worldwide prevalence of 1–2% and a worldwide economic burden accounting for USD 108 billion annually, this disease affects the lives of many [4,5]. Treatment strategies targeted at a reduction in cardiac energy consumption have decreased morbidity and mortality over the past several decades [6]. Nonetheless, even after therapeutic optimization according to the most recent guidelines, impaired exercise capacity continues to be one of the most impactful symptoms of HF [1,2,3]. Table 1 provides an overview of the effect of guideline-recommended cardiovascular agents on exercise tolerance in HF. Although these therapeutic options have several beneficial effects on cardiac failure, the summary of these data underlines the fact that exercise intolerance in HF remains difficult to treat to this day. Exercise intolerance in HF is multifactorial in origin with underlying cardiovascular pathophysiological mechanisms as well as changes in skeletal muscle tissue and metabolism.

Developments in the field of sodium-related glucose transporter (SGLT) 2 inhibitors have recently led to compelling changes in the international HF guidelines [3,26,27]. Remarkably, it was safety trials in type 2 diabetes mellitus (T2DM) patients that brought to light the drugs’ unexpected benefits for HF; the EMPA-REG OUTCOME, CANVAS, DECLARE-TIMI58, and VERTIC-CV trials showed that empagliflozin, canagliflozin, dapagliflozin, and ertugliflozin, respectively, reduce the risk of cardiovascular mortality and HF hospitalizations in patients who are at cardiovascular risk [26,28,29,30]. These randomized controlled trials provided the cornerstones for the initiation of HF-targeted SGLT2 inhibitor trials. The DAPA-HF and EMPEROR-REDUCED trials were the first to show that the beneficial effects on cardiovascular endpoints in stable HF with reduced ejection fraction (HFrEF) existed independently of the presence of T2DM [31,32]. Additionally, the CANONICAL, EMPEROR-PRESERVED, and DELIVER trials were initiated to evaluate the effect of SGLT2 inhibition in HF with preserved ejection fraction (HfpEF) [33,34,35]. Recently, the EMPEROR-PRESERVED trial was the first to show that empagliflozin significantly reduced cardiovascular endpoints in HfpEF, making it one of the first effective and evidence-based medical therapies for the treatment of the type of HF that accounts for approximately 50% of the total HF population [36,37]. Completing the spectrum, the potential benefits in patients with acute HF [38,39,40] and patients at risk for HF after acute myocardial infarction [41,42] were investigated in the SOLOIST-WHF, DICTATE-HF, EMPULSE, and EMPACT-MI trials, respectively. The results of the SOLOIST-WHF and EMPULSE trials, showing clinical benefits after (semi-)acute hospitalization for HF, suggest that indications for SGLT2 inhibitors could extend to even more patients in the near future [40,43].

SGLT1 and SGLT2 are membrane transporter proteins that facilitate glucose reabsorption in different end-organs. While SGLT1 proteins are predominantly located in the small intestine, SGLT2 proteins are located in the proximal tubule of the nephron, where they are responsible for 90% of the glucose reabsorption in the kidney [44]. SGLT2 inhibitors work by blocking these SGLT2 transporters, promoting glycosuria, and modestly decreasing plasma glucose levels [45]. In response, a reduction in insulin concentration in combination with an increase in glucagon has been observed [45]. Although the first report on phlorizins was published in 1835 [46], it was not until the 21st century that SGLT inhibitors were developed further as antidiabetic drugs. However, since the beneficial cardiovascular effects of SGLT2 inhibitors in patients with T2DM were found to be primarily driven by a reduction in HF hospitalizations and the cardiovascular benefits exist independently of T2DM [31,32], the glycosuria effect seems to be too modest to explain the magnitude of the cardioprotective effect by itself [47,48,49,50]. Although many other physiological effects of SGLT2 inhibitors have been observed and many hypotheses have been suggested, the exact mode of action of these drugs remains incompletely understood.

In Europe, four different types of SGLT2 inhibitors have been approved for clinical use in T2DM patients who are at cardiovascular risk: dapagliflozin (Forxiga; Astra Zeneca, Cambridge, UK), empagliflozin (Jardiance; Boeringher Ingelheim, Ingelheim am Rhein, Germany), canagliflozin (Invokana; Janssen Research & Development, LLC, Raritan, NJ, USA), and ertugliflozin (Steglatro; Pfizer, New York, NY, USA) [51,52,53,54]. In Japan, ipragliflozin (Suglat; Astellas Pharma Inc., Tokyo, Japan), luseogliflozin (Lusefi; Taisho Pharmaceutical, Tokyo, Japan), and tofogliflozin (Apleway/Deberza; SANOFI/Kowa Pharmaceuticao) have also been approved for the treatment of T2DM [55,56,57]. Furthermore, sotagliflozin (Zynquista; Sanofi and Lexicon pharmaceuticals), which is an SGLT1/SGLT2 inhibitor, has been approved for the treatment of type 1 diabetes mellitus [40]. Empagliflozin and dapagliflozin are currently recommended for the treatment of HfrEF, regardless of the presence of T2DM [27].

### 1.2. Exercise Intolerance in HF

Despite continuous developments in therapeutic treatment options for HF, exercise intolerance remains a problem in many patients (Table 1). Exercise intolerance is characterized by a disproportionate early onset of fatigue in relation to the intensity, duration, and type of the activity that is being performed [1,2,3,58]. This leads to impairment of the quality of life and physical functioning of HF patients on a daily basis and is associated with increased mortality [3,59,60,61,62]. Although symptoms of dyspnea during rest can remain absent until the most severe disease stage (NYHA IV), the slightest increase in activity level can provoke symptoms in early stages of HF. With an average peak VO2 of 10–20 mL/kg/min, HF patients have a ±35% decrease in maximum oxygen uptake as compared with age-matched controls [62,63,64,65]. In addition, multiple studies have shown HF-induced pathophysiological changes in skeletal muscle. HF is associated with a reduction in maximal muscle mass, a reduction in muscle strength, and impairment of oxidative skeletal muscle metabolism [66,67,68,69].

Improving exercise tolerance by exercise therapy is a safe and effective way to improve quality of life in HF patients [70,71]. In fact, exercise training programs are now highly recommended in the recently updated European guidelines on sports cardiology and exercise in patients with stable HF [72]. However, implementation of these exercise programs remains a challenge within this patient population, which is characterized by a high prevalence of comorbidities that influence physical functioning. While many studies on the effect of SGLT2 inhibitors on cardiovascular endpoints have been conducted, little is known about their effect on exercise capacity in HF patients, emphasizing the need to evaluate the potential effects of SGLT2 inhibitors on exercise tolerance in HF patients. This review: (1) elaborates on the pathophysiology of exercise intolerance in HF; (2) discusses the potential modes of action of SGLT2 inhibitors on these phenomena; and (3) provides an up-to-date overview of the available studies on SGLT2 inhibitors and their effect on clinical outcome parameters, including physical functioning and exercise capacity. Finally, we also (4) elaborate on gaps in the evidence and potential future perspectives on the effects of SGLT2 inhibitors on exercise intolerance in chronic heart failure.

## 2. Pathophysiology of Exercise Intolerance in Heart Failure

**Figure 1:**
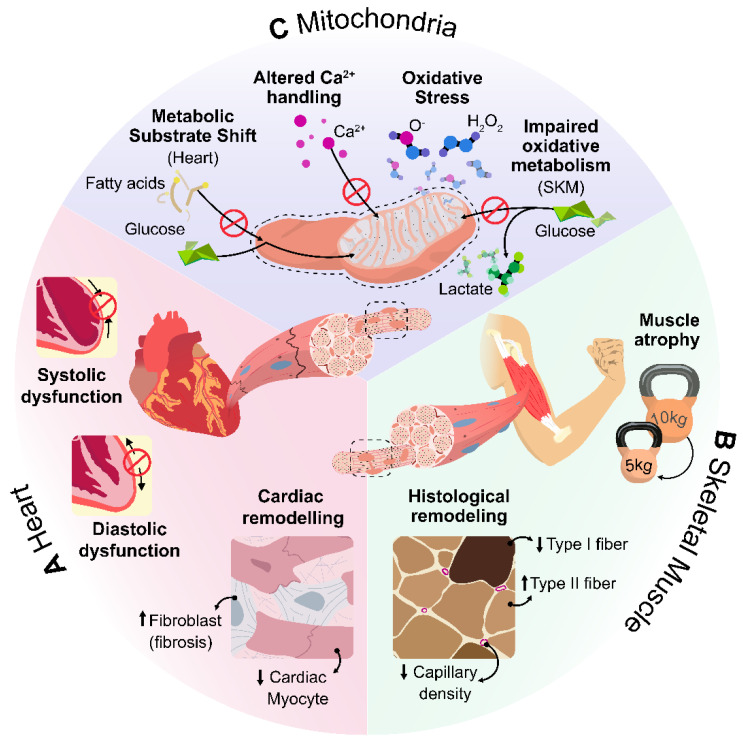
Pathophysiological mechanisms underlying exercise intolerance and their interactions in HF. In the multifactorial origin of heart failure, pathophysiological changes in several organ systems contribute to the development of signs and symptoms. Exercise tolerance in HF is influenced by changes in: (**A**) impaired cardiovascular performance, including systolic dysfunction, diastolic dysfunction, and remodeling of the cardiac tissue; (**B**) changes in skeletal muscle, including histological remodeling of skeletal muscle and a reduction in skeletal muscle mass and strength; and (**C**) metabolic and mitochondrial changes, including mitochondrial dysfunction, impairment of mitochondrial calcium handling, an increase in oxidative stress, and impaired oxidative skeletal muscle metabolism.

In the multifactorial origin of HF, pathophysiological changes in several organ systems contribute to the development of signs and symptoms. Exercise tolerance in HF is influenced by changes in cardiovascular performance (Figure 1A), skeletal muscle (Figure 1B), and mitochondrial metabolism (Figure 1C) [2,59,73].

### 2.1. Impaired Cardiovascular Performance

The syndrome of HF is believed to develop as a two-step process starting primarily with underlying structural and/or functional cardiac abnormalities that result in systolic and/or diastolic cardiac dysfunction [3,74]. Initial cardiac dysfunction contributes to the onset of exercise-related symptoms and can be caused by damage to or dysfunction of the myocardial tissue or by abnormal loading conditions [3,75]. Systolic dysfunction leads to impairment of the left ventricular ejection fraction (LVEF) and characterizes HFrEF, which is often caused by ischemic heart disease, genetic abnormalities, or toxic damage [3]. HFpEF, on the contrary, is more often characterized by diastolic dysfunction in the form of impaired relaxation either with or without increased filling pressures [76]. Although the pathophysiological mechanisms of HFpEF remain incompletely understood, several cardiovascular and environmental risk factors are known to play an important role in its development, including hypertension, inflammation, obesity, and diabetes mellitus [36].

When systolic cardiac dysfunction persists, several systemic processes are activated that lead to remodeling of the cardiac tissue in the form of interstitial fibrosis or left ventricle hypertrophy [74,77,78]. Remodeling is caused by structural changes in the morphology of cardiac tissue due to loss of (functioning) cardiomyocytes and changes in the extracellular matrix, causing further deterioration of cardiac performance [74]. Interstitial fibrosis is characterized by an accumulation of excess fibrous tissue, such as collagen, in the myocardial interstitium [78]. This fibrous tissue can cause increased stiffness, decreasing myocardial mechanical performance. Hypertrophy and/or dilatation of the left ventricle can, on the other hand, lead to changes in the ventricle’s geometry, such as an increase in end-diastolic volumes, which in turn can decrease cardiac efficiency [77,79]. Several molecular pathways have been proposed to further contribute to cardiac remodeling, including changes in insulin signaling pathways [80,81].

Moreover, persistent cardiac dysfunction induces changes in myocardial fuel utilization [82,83,84]. To maintain its unremitting mechanical function, the myocardial tissue is highly dependent on a constant rate of ATP synthesis within cardiac mitochondria [85,86]. In a healthy heart, over 95% of the cardiac ATP is produced through oxidative phosphorylation [82]. Fatty acids (FAs) provide the vast majority of utilized substrates, while the remaining part is compounded by carbohydrate, lactate, and ketone bodies [82,83,87,88]. Nevertheless, the heart is a metabolically flexible organ that is able, if necessary, to shift to the utilization of other fuels depending on their availability in the circulation. In HF, energy substrate metabolism changes and metabolic flexibility decreases. The contribution of FA oxidation is attenuated [82,83,84] and the heart switches to other available fuels to keep cardiac ATP production as high as possible [83,87,89,90]. Remarkably, cardiac ATP levels can be sustained successfully until the end-stage disease state by several compensatory measures; an increase in ATP production through anaerobic phosphorylation has been detected in HF [84,86] in addition to a switch of substrate utilization in oxidative phosphorylation in the form of an increase in oxidation of the non-lipid-substrate ketone bodies, lactate, and amino acids [87,89,91,92,93,94]. Although cardiac ATP output can be sustained by this compensatory upregulation of anaerobic glycolysis, this switch in energy systems also leads to an increase in pathological remodeling of cardiac tissue [86]. The increased cardiac utilization of ketone bodies in oxidative phosphorylation, on the other hand, has been suggested to be an adaptive mechanism [90,95]. Ketone bodies are metabolites that are produced in liver hepatocytes and derived from FAs or triacylglycerol. Under conditions of absolute or relative nutrient deprivation, e.g., during fasting, prolonged exercise, or metabolic disease, hepatic ketone production is upregulated and ketone bodies can provide an energy source for extrahepatic organs such as the heart [96].

### 2.2. Changes in Skeletal Muscle

Although cardiac dysfunction is associated with exercise intolerance, it has been shown that the decrease in exercise capacity cannot be linearly explained by cardiac dysfunction. In response to the underlying cardiac pathophysiology, chronic HF manifests itself as a systemic disease in the skeletal muscle, impairing skeletal muscle function [2,64,97,98]. The finding that exercise intolerance in patients with HF persists independent of (recovery of) cardiac function supports the hypothesis that mechanisms independent of cardiac dysfunction further contribute to exercise intolerance in HF [64,99].

In multiple smaller-sized studies, biopsies from the vastus lateralis muscle provided evidence of histological remodeling of skeletal muscle in both HFrEF and HFpEF [98,100,101,102]. In both types of HF, the type I (oxidative) to type II (glycolytic) muscle fiber type ratio was decreased. The decrease in this ratio is most likely caused by an absolute decrease in type I fibers, although an increase in type II muscle fibers has also been reported [100]. However, in this specific study, the type II fiber size was smaller in HF as compared with that in healthy skeletal muscle tissue. While oxidative fibers display relatively high mitochondrial density and oxidative enzyme activity values, making them specialized in aerobic energy production, glycolytic fibers are specialized in ATP production through anaerobic glycolysis [103]. In the healthy population, this latter fiber type is characterized by speed and power but can easily be fatigued. In HF patients, the observed change in muscle fibers from the oxidative to the anaerobic type is associated with a reduction in exercise tolerance [100,102]. Several factors are believed to underlie this switch in fiber type, including an increase in oxidative stress and a decreased level of physical activity in this population [100,104,105]. The fact that exercise training can lead to a re-shift in the muscle fiber type distribution ratio in the HF population supports this theory and emphasizes the importance of physical activity in this population [106]. However, the changes that are seen in HF exceed the level of impairment that is solely caused by a sedentary lifestyle in healthy individuals [100,107]. Skeletal muscle in HF furthermore expresses a decrease in the capillary density surrounding each muscle fiber [100,102,108]. ln contrast to the aforementioned histological changes, these changes are HF-specific, as they were not detected in sedentary controls [100].

Patients with chronic HF display a reduction in skeletal muscle mass and strength [109]. One of the underlying factors is the loss of functional skeletal muscle tissue, i.e., sarcopenia, which has been demonstrated by muscle biopsies in several studies [67,102,110,111,112]. A strong correlation has been reported between muscle cross-sectional area and muscular strength [113]. Skeletal muscle atrophy and apoptosis of skeletal muscle myocytes have consistently been observed as frequent comorbidities in HF patients and are associated with exercise intolerance [66,102,110,111,112,114]. In both types of HF, sarcopenia contributes to the vicious cycle of deconditioning and deterioration of physical functioning [110]. In HFrEF, hormonal changes that are consistent with a catabolic–anabolic imbalance are believed to further contribute to the development of cardiac cachexia [115]. Several other underlying mechanisms have been proposed to influence the reduction in muscle strength, including impaired insulin sensitivity. Quadriceps muscle strength was strongly related to insulin sensitivity in chronic HF patients [109]. Because impaired insulin sensitivity is strongly related to both the onset and the pathophysiology of HF, this is likely of relevance to the development of exercise intolerance [86,116,117].

HF is associated with impaired oxidative skeletal muscle metabolism as shown by dynamic in vivo measurements with 31 phosphorus (31P) magnetic resonance spectroscopy (MRS) [2,68,69,118]. This technique, which allows for quantification of high-energy phosphate decline rates during exercise and post-exercise rates of recovery, provides an adequate method for assessing in vivo mitochondrial oxidative metabolism. Previous studies showed an increased depletion of highly energetic phosphates and a prolonged phosphocreatine (PCr) recovery rate, implying that oxidative mitochondrial function in skeletal muscle is impaired in HF during exercise [2,69,119,120]. A detected decrease in intramuscular pH during exercise [68,69] and increase in blood lactate levels [100] in HF compared with healthy subjects furthermore suggest that abnormal skeletal muscle metabolism induces an earlier shift from oxidative metabolism to glycolytic metabolism, as discussed below. The 31P MRS in vivo measurements have been shown to correlate well with in vitro measurements of mitochondrial capacity by muscle biopsies [121,122], supporting the conclusion that the rapid decline in high-energy phosphates and prolonged resynthesis rate in HF during exercise reflect impairment of mitochondrial oxidative capacity [2,121].

### 2.3. Metabolic and Mitochondrial Changes

One of the underlying pathophysiological mechanisms of mitochondrial dysfunction in HF is impairment of mitochondrial calcium handling [123]. Calcium handling regulates mitochondrial activity and is essential for muscular contraction and relaxation [123,124]. Calcium handling is regulated by the sarcoplasmic reticulum in the cell and the exchange of calcium across the sarcolemma cycle affects the time course and magnitude of the force output by the muscle fibers [125,126]. In HF, calcium release and reuptake over the sarcoplasmic reticulum are impaired due to decreased levels of well-functioning sarcoplasmic reticulum calcium ATPase (SERCA2a) [127]. Impaired calcium handling has been observed in both cardiomyocytes [127] and skeletal muscle [126,128], suggesting a common pathophysiological mechanism. Furthermore, impaired calcium handling was found to be associated with the early onset of fatigue in HF [129].

In addition, upregulation of inflammatory mediators and oxidative stress contribute further to the development of HF syndrome. In failing cardiomyocytes, oxidative stress has been found to be enhanced and to induce cardiac remodeling [130]. While there is a lot of evidence for this mechanism in the heart [74,130,131], there is little consensus on the question of whether this phenomenon occurs in skeletal muscle as well; while some studies suggest that local oxidative stress in skeletal muscle is increased in HF, contributes to the early onset of fatigue, and plays a role in muscle deterioration [97,132,133], others did not find proof of a contributing role of oxidative stress in HF-induced skeletal muscle changes [128].

As touched upon above, mitochondrial dysfunction in HF develops in both cardiomyocytes and skeletal muscle and results in impairment of ATP production [82,83,134]. Dynamic mitochondrial processes in the form of fusion, autophagy, and fission are crucial to the vitality and lifespan of mitochondria [135]. However, in HF mitochondrial dynamics are impaired [136]. One of the mechanisms responsible for the impediments in mitochondrial dynamics is the increased acetylation of mitochondrial proteins in the failing heart [86,135] as well as in skeletal muscle [134]. Increased protein acetylation interferes with mitochondrial dynamics and hinders mitochondrial metabolism. It can also aggravate myocardial calcium mishandling and oxidative stress, the two previously discussed phenomena that can further contribute to mitochondrial impairment [86,135]. Impaired mitochondrial dynamics lead to an eventual decrease in functional mitochondrial volume density [101]. In the end, this results in a vicious circle by inducing further deterioration of myocardial energy metabolism.

Another HF-induced change contributing to exercise intolerance is the observed shift in energy production from oxidative phosphorylation to anaerobic glycolysis. In the healthy state, cardiac function is predominantly dependent on oxidative phosphorylation for the generation of ATP. Oxidative phosphorylation is the most efficient system for energy production. Oxidative phosphorylation predominantly uses lipids as energy substrates and is dependent on well-functioning mitochondria [137]. When upregulation of mechanical activity results in a higher energy demand than mitochondria can generate through aerobic phosphorylation, muscle tissues shift to ‘non-mitochondrial’ anaerobic glycolysis for ATP production to sustain mechanical activity as long as possible [137,138]. By anaerobic glycolysis, carbohydrates can be converted from a fuel substrate to ATP without the use of oxygen. However, due to the increase in breakdown products, this energy production system leads to an earlier onset of fatigue as compared with oxidative phosphorylation. HF is characterized by a shift from aerobic FA oxidation to anaerobic glucose oxidation, both in cardiac and skeletal muscle, resulting in all the aforementioned consequences [68,69,100].

In HF, deconditioning further contributes to the development of exercise intolerance. Deconditioning can be a consequence of several factors, including the burden of disease symptoms, the high prevalence of comorbidities, and/or cardiovascular risk factors and unhealthy lifestyle habits, that contribute to the initial onset of HF [108,139]. Deconditioning accelerates many of the aforementioned pathophysiological changes, including the decrease in cardiac function, the loss of functional skeletal muscle, the shift in the muscle fiber type, and the shift in the energy production system away from oxidative energy production [111,119,140]. This can create a vicious circle of increases in the symptom burden and disease progression for HF patients and contributes to the symptoms of exercise intolerance as well.

### 2.4. Assessment of Exercise Capacity in HF

**Figure 2:**
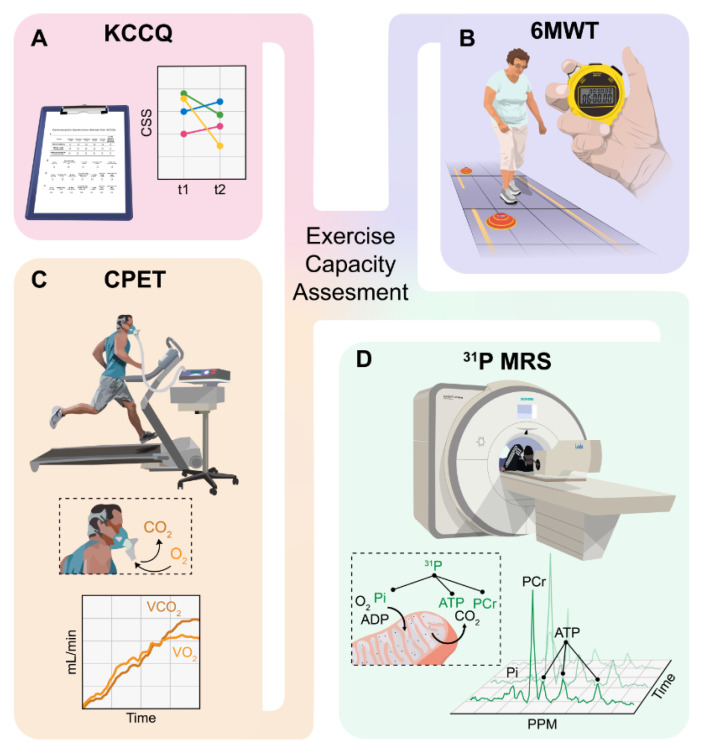
Different tools for the assessment of exercise capacity in heart failure. Abbreviations: (**A**) KCCQ, Kansas City Cardiomyopathy Questionnaire; CSS, clinical symptom score; (**B**) 6MWT, 6-min walk test; (**C**) CPET, cardiopulmonary exercise testing; (**D**) 31P MRS, 31 phosphorus (31P) magnetic resonance spectroscopy; ATP, adenosine triphosphate.

While there are several ways to assess exercise performance in HF, we will focus our analysis on the most robust and commonly used methods: (1) the Kansas City Cardiomyopathy Questionnaire (KCCQ) on self-reported physical function and quality of life; (2) the six-minute walk test (6MWT); (3) cardiopulmonary exercise testing (CPET) with maximal oxygen uptake (VO2) measurements; and (4) 31P MRS testing (Figure 2).

The KCCQ consists of 23 items and quantifies several factors that influence quality of life, including the clinical summary score (CSS), which is most representative of (self-reported) physical functioning (Figure 2A). This diagnostic tool is often used to quantify the impact of the disease on physical functioning and can be of prognostic use in HF. It is a tool that is cheap and easy to implement, and it has been shown that changes in KCCQ score are sensitive to clinical changes [141] and future risk in patients with HF [141,142]. However, it should be taken into account that this measure is a subjective diagnostic tool and results from it are only poorly correlated with other tools for the assessment of exercise capacity, such as the 6MWT or CPET [143,144].

The 6MWT is a walking test in which patients are encouraged to walk as far as possible through a corridor in a timeframe of six minutes (Figure 2B). There is evidence that this diagnostic tool has high reproducibility [145,146], and it was found that the results are a good reflection of moderate or large changes in clinical deterioration in the opinion of the cardiologist [141]. However, the question remains as to what extent results from this tool truly reflect exercise capacity. Because walking speed cannot be standardized, patients often start too fast or must take a break during the 6 min of a self-chosen duration, causing heterogeneous results. Furthermore, the 6MWT can be influenced significantly by comorbidities that influence walking speed.

To this day, CPET with VO2 measurement remains the gold standard for the assessment of exercise tolerance in HF (Figure 2C) [72,146,147]. HF patients generally reach a VO2 max of approximately 65% of that of age-matched controls [65,111]. This method provides an objective way to assess exercise tolerance in HF and can either be performed on a treadmill or on an ergometer [144,148]. Peak VO2, the most used CPET variable, reflects functional capacity and was found to predict mortality in HF [61]. The downsides of this method of testing are that it is expensive and time consuming. Moreover, subjects need to be able to perform an exercise test until exhaustion to obtain a reliable measurement of VO2 capacity. Lastly, just like the 6MWT, this diagnostic tool can also be influenced significantly by comorbidities and the measurement of metabolism is an indirect assessment.

Testing exercise capacity with 31P MRS has many advantages in comparison with the aforementioned tools (Figure 2D). This diagnostic method provides a direct and dynamic measurement of ATP production and mitochondrial function [121] and several studies have shown that it is a suitable method for assessing skeletal muscle oxidative capacity in HF [2,68,69,119]. One of the benefits is that it does not require the patient to achieve a maximal level of exertion to obtain information about muscle energetics. Furthermore, this assessment provides a direct reflection of metabolism in contrast to the other tools. However, just like the CPET, the 31P MRS is an expensive and time-consuming method. To our knowledge, no studies have assessed the effect of SGLT2 inhibitors on exercise metabolism using this technique.

## 3. SGLT2-Inhibition-Mediated Effects on the Underlying Pathophysiological Mechanisms of Exercise Intolerance

**Figure 3:**
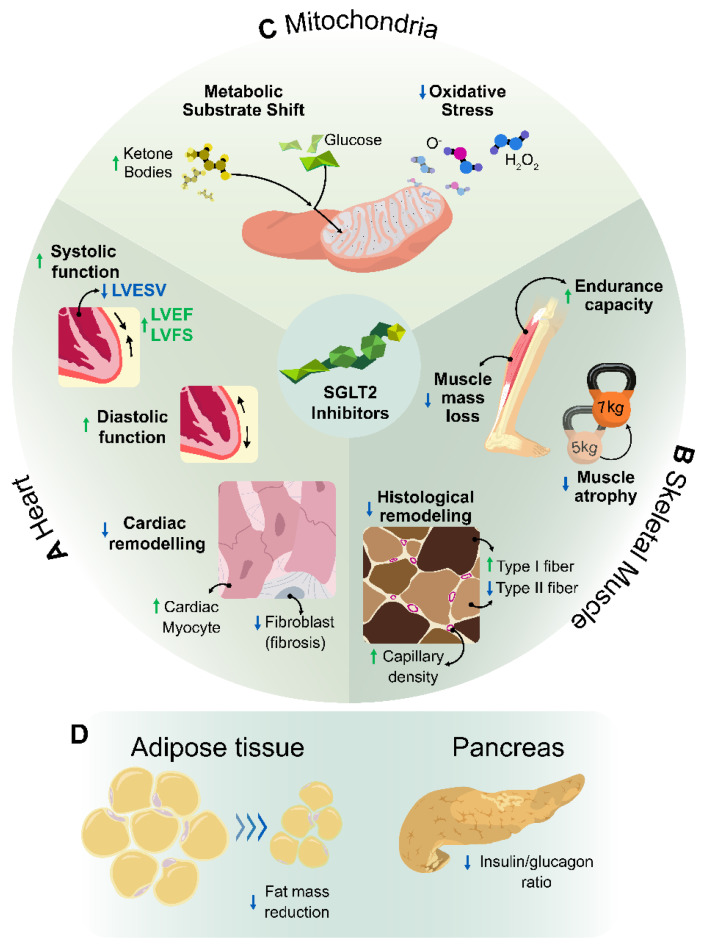
Potential effects of SGLT2 inhibitors within different organ systems that influence exercise capacity in HF beneficially. This figure depicts potential benefits of sodium-related glucose transporter 2 (SGLT2) inhibitor treatment on the heart, skeletal muscle, and metabolism, potentially improving exercise tolerance in heart failure. These benefits include: (**A**) improvement of cardiovascular performance, including improvement of systolic function, improvement of diastolic function, and attenuation of cardiac remodeling; (**B**) improvement of skeletal muscle function, including amelioration of skeletal muscle remodeling, an increase in muscle strength, preservation of skeletal muscle mass, and improvement of muscle endurance capacity; (**C**) beneficial metabolic and mitochondrial effects, including improvement of mitochondrial function, a metabolic substrate shift leading to a beneficial change in cardiac energy production, and a decrease in oxidative stress; (**D**) a reduction in bodyweight and a decrease in the insulin–glucagon ratio.

A wide range of effects on cardiovascular performance, skeletal muscle tissue, and metabolism have been described as a response to treatment with SGLT2 inhibitors. The following paragraphs and Figure 3 provide an overview of the potential benefits of SGLT2 inhibition on exercise performance from experimental and clinical research in models of cardiovascular disease.

### 3.1. Effect of SGLT2 Inhibitors on Cardiovascular Performance 

The observed effects of empagliflozin on systolic function in diabetic mouse models are ambiguous. While some studies showed no improvement after empagliflozin treatment [149,150], others found an improvement in LVEF or fractional shortening after empagliflozin treatment [151,152]. In experimental HFrEF models, SGLT2-inhibitor-mediated effects on systolic function are more conclusive, as both empagliflozin and dapagliflozin have been shown to improve LVEF in multiple studies [138,153,154]. Out of three clinical studies on the effects of SGLT2 inhibitors in cardiovascular disease patients as measured on cardiac MRI (EMPA-HEART, including T2DM patients with coronary artery disease; Sugar-DM-HF, including T2DM-HFrEF patients; and EMPA-TROPISM, including non-diabetic HFrEF patients), only the EMPA-TROPISM study found evidence of SGLT2-inhibitor-mediated improvements in LVEF [155]. Regarding changes in end systolic volume (LVESV), two studies found a decrease in LVESV after treatment with empagliflozin for six or nine months [155,156], while others found no significant effect after three months of treatment with empagliflozin [157] or one year of treatment with dapagliflozin [158].

Regarding diastolic function, the results from the effect of empagliflozin are more uniform. Several diabetic mouse models showed that treatment with empagliflozin had a beneficial effect on diastolic cardiac function [149,150,151]. In isolated muscle fibers from human end-stage failing hearts, a wash-in treatment protocol with empagliflozin led to an improvement in diastolic function [159]. In animal models of both HFrEF and HFpEF, treatment with empagliflozin and dapagliflozin, respectively, led to an improvement in diastolic function [160,161]. In two clinical cardiovascular cohorts with T2DM patients, diastolic function improved after 3 months and 6 months of treatment with empagliflozin without changing end diastolic parameters [157,162]. Furthermore, a clinical study in non-diabetic HFrEF patients based on echocardiographic results showed mitigation in end diastolic parameters after 6 months of treatment with empagliflozin [155] and also after treatment with canagliflozin [163].

In diabetic [149,151] as well as HF animal models [153,160], treatment with empagliflozin led to attenuation of cardiac remodeling by decreasing LV hypertrophy and reducing interstitial cardiac fibrosis. In addition, several clinical studies including T2DM patients with coronary artery disease and/or HFrEF patients all found significant reductions in the LV mass index after treatment with empagliflozin [155,156,157,162], suggesting that empagliflozin could ameliorate LV hypertrophy in the clinical setting as well. In regard to dapagliflozin, one study found attenuation of fibrosis in a diastolic rodent model of HF [161], while in HF patients with T2DM no beneficial effects on LV remodeling were found [158].

It has been observed on a large scale and in both experimental and clinical settings that treatment with SGLT2 inhibitors endogenously increases circulating ketone bodies, potentially changing myocardial substrate utilization [45,138,153,158,164]. It has been reported that cardiac ketone utilization is already upregulated in HF [87,89,92]. Furthermore, two different transgenic mouse models have shown that this increase in cardiac ketolysis has a cardioprotective effect on delaying pathological remodeling in the failing heart [90,165]. Ketone bodies can readily be used as an energy substrate for cardiac ATP production in HF [87,94], suggesting that SGLT2 inhibitors could target the myocardial energy deficit that is caused by the altered cardiac fuel use. It has been shown in experimental models that empagliflozin improves cardiac ATP production in HF [138,153] and that upregulation of ketone utilization could likely contribute to this effect. Although the mechanism behind upregulation of ketone bodies by SGLT2 inhibitors is incompletely understood, it has been suggested that the reduction in the insulin–glucagon ratio induces an increase in hepatic FA oxidation, which leads to an increase in ketogenesis [48].

Furthermore, improved intracellular calcium handling could be one of the processes underlying the SGLT2-inhibitor-induced improvements in cardiac function [47,166]. HF is associated with an overexpression of the sodium–hydrogen exchanger (NHE), a translocator in the plasmatic membrane that exchanges sodium for protons [47]. This overexpression leads to an increased efflux of intracellular calcium and a lower concentration of calcium in the sarcoplasmic reticulum [123,124]. One of the off-target effects of SGLT2 inhibitors is inhibition of the NHE, through which SGLT2 inhibitors are hypothesized to restore calcium handing in the cardiomyocytes [47,166]. Several experimental models provided proof of this hypothesis, showing that both empagliflozin and dapagliflozin influence calcium handling through lowering NHE activity in cardiomyocytes from rabbits or rats [150,161,167]. In contradiction, however, other authors found that calcium homeostasis in human failing cardiomyocytes was not influenced by empagliflozin treatment in cultured human cardiomyocytes [159]. Moreover, multiple studies failed to detect reduced NHE1 activity upon empagliflozin treatment [168] or did not find differences in calcium ATPase protein expression after empagliflozin treatment [149].

### 3.2. Effect of SGLT2 on Skeletal Muscle

It has been suggested that SGLT2 inhibition could ameliorate the remodeling of skeletal muscle. In a mouse model with reduced aerobic exercise capacity due to chronic hyperglycemia, treatment with canagliflozin led to an increase in the proportion of oxidative fibers, suggesting that impaired muscle remodeling was prevented by treatment with SGLT2 inhibitors [169]. In a high-fat diet mouse model in which mice were treated with canagliflozin for 8 weeks, SGLT2 inhibition induced a reduction in inflammatory cytokine levels and macrophage accumulation [170]. Decreasing oxidative stress or inflammation could have a beneficial effect in preventing a shift in muscle fiber type in skeletal muscle [170]. Furthermore, treatment with canagliflozin was found to restore exercise-induced angiogenesis in mice that were exposed to hyperglycemia [169]. The question of whether SGLT2 inhibitors could have similar effects on amelioration of the HF-induced remodeling in skeletal muscle has not yet been studied.

It has been hypothesized that SGLT2 inhibitors might reverse skeletal muscle atrophy, making them potential drugs that target muscle strength [171]. Several underlying pathways for targeting muscle strength have been proposed, including an increase in the expression of FOXO1 [172] and a decrease in hyperglycemia, which promotes skeletal muscle atrophy through the WWP1/KLF15 pathway [173]. Another potential pathway is the improvement of insulin signaling, which has been observed after SGTL2 inhibition treatment in diabetic mice [174] and patients [175]. However, results on the effect on muscle strength are contradictory; while grip strength significantly improved in non-HF diabetic rodents treated with luseogliflozin [171], treatment with empagliflozin in mice with HF showed no increase in muscle strength [176]. A study in 112 diabetic patients without HF treated with either ipragliflozin, luseogliflozin, or dapagliflozin demonstrated that patients’ hand grip strength increased after treatment with all three SGLT2 inhibitors [177].

Several trials have investigated the effect of SGLT2 inhibition on skeletal muscle mass. In obese mice without HF, skeletal muscle weight was unaffected by six weeks of treatment with empagliflozin [178]. In diabetic, non-HF subjects, treatment with luseogliflozin led to a significant increase in soleus muscle weights in rodents, while skeletal muscle mass remained unaffected in the human population treated with canagliflozin or dapagliflozin for six months [179,180]. Another study in T2DM patients even showed a small decrease in skeletal muscle mass after luseogliflozin treatment for 36 weeks [181]. Recently, Otsuka et al. showed that, in healthy mice treated with canagliflozin, grip strength was unaffected during ad libitum feeding while a significant reduction was present when mice were pair-fed in the same amounts as vehicle-treated controls [182]. Interestingly, glycolytic type II muscle fiber mass and function were affected, while oxidative type I muscles remained unaffected.

As previously stated, it has been shown in both experimental and clinical models that SGLT2 inhibitor treatment upregulates ketone bodies. The effects of ketone bodies on skeletal muscle are different as compared with the effects on the heart. Ketolytic enzymes such as succinyl-CoA:3-ketoacid CoA transferase (SCOT) are expressed in lower rates in the skeletal muscle as compared with the myocardium [183,184]. Although skeletal muscles have a high affinity for ketone bodies, they provide less than 5% of the fuel substrates in healthy, resting muscles [183]. During exercise, however, ketone bodies can become the preferred fuel source in skeletal muscle [185,186]. In a healthy mouse model treated with intravenous infusion of ketone bodies, it was shown that sensitivity to ketone bodies was higher in skeletal muscle with a glycolytic phenotype [187]. Because of the HF-induced shift from the oxidative phenotype to the glycolytic phenotype of skeletal muscle, this suggests that ketone bodies could provide a potential benefit for skeletal muscle metabolism in HF. Interestingly, in HF, it was found that the upregulation of cardiac ketone utilization was accompanied by a downregulation in skeletal muscle, as was measured in the fraction of ketone bodies that were extracted from arterial blood [89]. It remains unknown whether this downregulation is caused by competitive fuel use of the myocardium and whether exogenous suppletion of ketones could further enhance oxidative metabolism in skeletal muscle in HF.

In murine ischemic HF models, empagliflozin treatment improved muscle endurance capacity in HF [176,188]. In an ischemic HF model, empagliflozin treatment led to an increase in mitochondrial respiration in skeletal muscle [188]. Two studies found evidence of an amelioration of the HF-induced decline in the four-limb hanging time in relation to bodyweight after treatment with empagliflozin [176,188]. To our knowledge, the effect of SGLT2 inhibitors on oxidative muscle metabolism in HF patients has not been assessed. Interestingly, in streptozotocin-treated mice, SGLT2 inhibitor treatment led to a restoration of the training response [169]. In this study, mice were treated with canagliflozin for 8 weeks and exercise capacity was assessed before and after 6 weeks of voluntary wheel running. After correction for lean body mass, improvements in VO2 peak values were only observed in exercise-trained mice and not in sedentary controls. If these findings could also be reproduced in a clinical study, the potential of SGLT2 inhibition in combination with exercise training as a target to improve exercise capacity could be further investigated.

### 3.3. Metabolic and Mitochondrial Effects of SGLT2 Inhibitors

In addition to the effect on mitochondrial fuel substrate availability, SGLT2 inhibitors can improve mitochondrial function through several other mechanisms. Different studies showed that SGLT2 inhibition led to a decrease in mitochondrial DNA damage in HF [153,189]. Furthermore, a decrease in oxidative stress has been found after treatment with SGLT2 inhibitors [86].

Several studies in diabetic and HF rodents have showed that SGLT2 inhibitors suppressed oxidative stress in both cardiac and skeletal muscle tissue [153,170,190,191,192]. One of the potential mechanisms behind this decrease in oxidative stress is the increase in ketone bodies, which reduce oxidative stress and insulin resistance in the skeletal muscle [183].

Treatment with SGLT2 inhibitors positively influences one of the causes of deconditioning by inducing a significant reduction in bodyweight (Figure 3D). In diabetic rodents, treatment with luseogliflozin led to a significant loss of weight [171]. In human subjects, treatment with empagliflozin led to an average weight loss of 3.2 ± 4.2 kg despite an increased caloric intake [193], and comparable results were found after treatment with dapagliflozin [194], after treatment with canagliflozin [195], and in a retrospective cohort treated with a mixture of SGLT2 inhibitors [196]. The study by Sakatomo et al. showed that SGLT2 inhibitors reduced bodyweight despite no change in skeletal muscle mass [196]. Other studies showed that SGLT2-inhibition-induced weight loss was primarily driven by a reduction in fat mass [195] and by reducing both visceral and subcutaneous fat tissue [178,180]. Weight loss in general might be beneficial in treating exercise intolerance, as obesity is a common comorbidity of HF that influences exercise tolerance negatively [139], and exercise capacity is expressed in terms of maximal oxygen uptake in relation to body mass. Moreover, a growing body of evidence suggests that treatment with SGLT2 inhibitors might decrease epicardial fat in T2DM patients [180,197] and HF patients [198]. This could be another potential factor that contributes to improving exercise tolerance by improvement of diastolic function.

SGLT2 inhibition induces a beneficial change in cardiac energy production by shifting fuel utilization from (anaerobic) glycolysis to lipid oxidation [45,138,199]. This was measured with calorimetry in experimental models with obese mice [178], mice with HF [176], and pigs with HF [138] that were all treated with empagliflozin. These findings were supported by results from a clinical study in T2DM patients showing that dapagliflozin led to a shift in energy metabolism from glucose oxidation to fat oxidation [164]. One of the mechanisms behind this re-shift could be the upregulation of ketone bodies that is induced by SGLT2 inhibition. Ketone oxidation can only produce ATP through oxidative phosphorylation, and cardiac ketone utilization is determined by substrate availability [83]. The increase in ketone bodies could lead to a downregulation of anaerobic glycolysis and an accompanying downregulation in the accessory negative effects of glycolysis, such as increased oxidative stress and impairment of intracellular calcium handling [86,135].

## 4. Effect of SGLT2 Inhibitors on Exercise Performance in HF

Table 2 provides an overview of the studies on SGLT2-inhibitor-induced effects on exercise intolerance in HF that were conducted using the tools for assessment described above.

### 4.1. Effects of SGLT2 Inhibitors on Self-Reported Physical Performance (KCCQ)

Results on the SGLT2-inhibitor-induced effects on KCCQ are well-investigated but very heterogeneous in nature. Empagliflozin treatment did not lead to an improvement in KCCQ-CSS after 3 months of treatment in HFrEF in the EMPIRE-HF trial [200] nor after 8 months of treatment in the SUGAR-DM-HF trial in diabetic patients with HFrEF [156]. In addition, the EMPERIAL trial found no significant changes in KCCQ after 3 months of treatment in both HFrEF and HFpEf patients [201]. It should be borne in mind, however, that the primary endpoint of these studies was neutral, making this analysis exploratory in nature [201]. In contrast, in the KCCQ substudy of the EMPEROR reduced trial, KCCQ-CSS was significantly improved after 3 months of treatment [202]. In addition, the EMPA-TROPISM trial found a significant improvement in the KCCQ-12 score with empagliflozin [155]. Regarding dapagliflozin, the results are scarcer but more uniform. While the DEFINE-HF trial showed a significant improvement in KCCQ-CSS after 3 months of treatment, these effects were not present after 6 weeks [203]. Moreover, the DETERMINE reduced trial and the KCCQ substudy of the DAPA-HF trial showed that from 3 months of treatment onwards, significant improvements in KCCQ-CSS can be detected [204,205]. The effect of 3 months of treatment with canagliflozin is currently being investigated by the CHIEF-HF trial [206].

### 4.2. Effects on Walking Distance (6MWT)

The EMPA-TROPISM trial, including 84 patients with an LVEF of <50%, found a significant improvement in 6-min walking distance (6MWD) after 6 months of treatment with empagliflozin [155]. Similar results were obtained in a small and non-placebo-controlled study with 19 symptomatic HF patients with T2DM, who were treated with empagliflozin for just 1 month [207]. However, the EMPERIAL trial, which was adequately powered for 6MWT, did not detect a significant effect of empagliflozin on 6MWD in the non-diabetic HFrEF or HFpEF population [201]. Neither did the SUGAR-DM-HF trial, which investigated the effect of 8 months of treatment with empagliflozin in diabetic HFrEF patients [156]. In line with these results, the DETERMINE reduced trial also found no significant increase in walking distance after 4 months of treatment with dapagliflozin in HFrEF patients [204]. The results on the effect of 4 months of treatment with dapagliflozin from the DETERMINE preserved study will be made available in the near future [208].

### 4.3. Effect on Cardiorespiratory Performance (CPET with VO2 Max)

Most of the CPET studies that have been performed in the context of SGLT2 inhibition are non-placebo-controlled pilot studies. The first two pilot studies assessing the effect of SGLT2 inhibitors with CPET in the human population investigated the effect of treatment with empagliflozin for one month in HFrEF patients with T2DM in a non-placebo-controlled study protocol [207,209]. These studies did not provide uniform results. While the study by Núñez et al. showed an increase in peak VO2 of 1.21 mL-kg/min in 19 patients [207], Carbone et al. did not find a significant improvement in peak VO2 in 15 patients [209]. The third pilot study, CANA-HF, which investigated the effect of 3 months of treatment with canagliflozin in a non-placebo-controlled small trial that was performed in order to compare canagliflozin to sitagliptin, also did not find a significant difference in peak oxygen consumption after 3 months of treatment [210]. Kumar et al. included 20 T2DM patients at high risk of cardiovascular disease and found that treatment with empagliflozin for 3 to 6 months significantly increased peak VO2 compared with usual medical care [211]. The only large, randomized, and controlled trial that has been performed is the EMPA-TROPISM trial, which randomized 84 non-diabetic HFrEF patients to treatment with empagliflozin or treatment with a placebo for 6 months, after which a CPET test with VO2 peak measurement was performed [155]. Interestingly, both the peak O2 consumption slope and the oxygen uptake efficiency slope were significantly improved in the group treated with empagliflozin as compared with placebo-treated patients. It should be noted, however, that the EMPA-TROPISM trial is relatively small compared with similar studies that used VO2 max as an endpoint. As such, the difference may have resulted from chance, and it is possible that the results may be different in other populations and with other compounds.

**Table 2:** Overview of clinical studies on the effect of SGLT2 inhibitors on exercise performance in HF.

Author	Trial Name	Year	Study Population	DM (%)	Intervention	Study Duration	Outcome Measure	Outcome KCCQ-CSS	Outcome 6MWT	Outcome CPET	Effect on Exercise
Kosiborod [205]	**DAPA-HF** **substudy**	2020	4443 HFrEF patients; NYHA II-IV; (LVEF ≤ 40%)	42%	Dapagliflozin 10 mg vs. placebo	8 months	KCCQ-CSS	KCCQ-CSS after 8 months: Increase ≥ 5 points: 1.18 (1.10 to 1.26); *p* < 0.0001. Increase ≥ 10 points: 1.19 (1.12 to 1.26); *p* < 0.0001. Increase ≥ 15 points: 1.14 (1.08 to 1.21); *p* < 0.0001.			Significantly improved KCCQ-CSS score after 4 and after 8 months of treatment
Butler [202]	**Substudy EMPEROR R**	2021	3730 HFrEF patients; NYHA II-IV; (LVEF ≤ 40%)	54%; 48%; 48% for patients with KCCQ of <62.5; 62.6–85.4 or >85.4 at baseline respectively	Empagliflozin 10 mg vs. placebo	12 months	KCCQ-CSS	KCCQ-CSS after 12 months: Increase ≥ 5 points: 1.22 (1.05 to 1.41); *p* = 0.0001. Increase ≥ 10 points: 1.22 (1.06–1.40); *p* = 0.0132. Increase ≥15 points: 1.17 (1.01–1.35); *p* = 0.0099.			Significantly improved KCCQ-CSS score after 3, 8 and 12 months of treatment
Nassif [203]	**DEFINE HF**	2019	263 HFrEF patients; NYHA II-III; (LVEF ≤ 40%)	62%	Dapagliflozin 10 mg vs. placebo	3 months	KCCQ-CSS	KCCQ-CSS: Increase ≥ 5 points: 2.4 (1.31 to 4.2); *p* < 0.01.			Significant improved KCCQ-CSS score
Spertus [206]	**CHIEF-HF**		1900 HF patients; HFrEF (LVEF ≤ 40%) or HfpEF (LVEF > 40%)		Canagliflozin 100 mg vs. placebo	3 months	KCCQ-CSS	NYP			NYP
Jensen [200]	**EMPIRE-HF**	2020	190 HfrEF patients; NYHA I-III; (LVEF ≤ 40%)	20% vs. 15% for patients treated with empagliflozin vs. placebo respectively	Empagliflozin 10 mg vs. placebo	3 months	KCCQ-CSS	KCCQ-CSS in adjusted difference of change (95% CI): 3.1 (−0.2 to 6.4); *p* = 0.07.			No significant change in KCCQ-CSS score
Abraham [201]	**EMPERIAL Reduced**	2021	312 HFrEF patients; NYHA II-IV; (LVEF ≤ 40%)	60%	Empagliflozin 10 mg vs. placebo	3 months	6MWT; KCCQ-TSS	KCCQ-TSS: Increase ≥ 8 points: 1.66 (1.02, 2.72)	Δ 6MWD was 13.5 [−8.0 to 42.0] meter after empagliflozin treatment vs. 18.0 [−11.5 to 54.0] meter after placebo treatment; *p* = 0.42.		No significant change in 6MWT No significant change in KCCQ-TSS
Abraham [201]	**EMPERIAL Preserved**	2021	315 HFpEF patients; NYHA II-IV; (LVEF > 40%)	51%	Empagliflozin 10 mg vs. placebo	3 months	6MWT; KCCQ-TSS	KCCQ-TSS: Increase ≥ 5 points: 0.98 (0.58, 1.63)	Δ 6MWD was 10.0 [−10.0 to 32.0] meter after empagliflozin treatment vs. 5.0 [−20.0 to 33.0] meter after placebo treatment; *p* = 0.37.		No significant change in 6MWT No significant change in KCCQ-TSS
Lee [156]	**SUGAR-DM-HF**	2020	105 patients with HFrEF + T2DM; NYHA II-IV; (LVEF ≤ 40%)	100%	Empagliflozin 10 mg vs. placebo	8 months	6MWT; KCCQ	KCCQ-TSS in between-group difference (95% CI): −4.0 (−10.2 to 2.1); *p* = 0.19.	Δ 6MWD was 25.4 ± 60.5 m after empagliflozin treatment vs. 33.6 ± 50.7 m after placebo treatment; *p* = 0.43.		No significant change in 6MWD
NYP [204]	**DETERMINE reduced**		313 HFrEF patients; NYHA II-IV; (LVEF ≤ 40%)	NYP	Dapagliflozin 10 mg vs. placebo	4 months	6MWT; KCCQ-TSS	KCCQ-TSS median score on a scale (IQR): 2.08 (−4.17 to 14.58) after dapagliflozin treatment vs. 0.00 (−10.42 to 9.38) after placebo treatment; *p* = 0.02164.	6MWD was 20.0 [−2.0 to 42.0] meter after dapagliflozin treatment vs. 13.5 [−12.5 to 46.5] meter after placebo treatment; *p* = 0.68626.		Significant improvement in KCCQ-TSS; No significant change in 6MWT
NYP [208]	**DETERMINE preserved**		504 HFpEF patients; NYHA II-IV; (LVEF > 40%)	NYP	Dapagliflozin 10 mg vs. placebo	4 months	6MWT; KCCQ-TSS				NYP
Carbone [209] ***		2018	15 patients with HFrEF * and T2DM; NYHA II-III; (LVEF < 50%)	100%	Empagliflozin 10 mg, not placebo-controlled	1 month	CPET (treadmill)			Peak VO2 changed from 14.5 [12.6–17.8] mL/kg/min at baseline to 15.8 [12.5–17.4] mL/kg/min after treatment; *p* = 0.95.	No significant change in peak VO2
Nuñez [207] ***		2018	19 patients with HF and T2DM; NYHA ≥ 2	100%	Empagliflozin 10 mg, not placebo controlled	1 month	CPET (ergometer)		Δ 6MWD after 1 month was 8.67%; *p* < 0.001.	Δ peak VO2 was +1.21 [0.66–1.76] mL/kg/min; *p* < 0.001	Significant improvement in peak VO2; Significant improvement in 6MWT
Santos-Gallego [155]	**EMPA-TROPISM**	2021	84 HFrEF * patients; NYHA II-IV; (LVEF < 50%)	0%	Empagliflozin 10 mg vs. placebo	6 months	CPET (ergometer); 6MWT; KCCQ-12	KCCQ-12 in Δ points ± sd: Difference from baseline 21 ± 18 vs. 1.9 ± 15; *p* < 0.001.	6MWD changed from 81 ± 64 m to −35 ± 68 m after 6 months; *p* < 0.001.	Δ peak VO2 was 1.1 ± 2.6 mL/kg/min in empagliflozin treated patients vs. −0.5 ± 1.9 mL/kg/min in placebo treated patients; *p* = 0.017.	Significant improvement in peak VO2; Significant improvement in 6MWT; Significant improvement in KCCQ-12
Carbone [210] ***	CANA-HF **	2020	36 patients with HFrEF and T2DM; NYHA II-III; (LVEF ≤ 40%)	100%	Canagliflozin 100 mg vs. sitagliptin 100 mg	3 months	CPET (treadmill)			Peak VO2 changed from 15.3 ± 3.5 mL/kg/min to 14.8 ± 3.9 mL/kg/min in sitagliptin treated patients; *p* = 0.16 and from 16.2 ± 3.4 mL/kg/min to 16.9 ± 4.0 mL/kg/min in canagliflozin treated patients; *p* = 0.23.	No significant change in peak VO2 after treatment with canagliflozin or sitagliptin.
Kumar [211] ***		2018	20 T2DM at high risk for cardiovascular disease (no HF)	100%	Empagliflozin 10 mg vs. usual care	3 to 6 months	CPET (ergometer)			Peak VO2 changed from 16.5 mL/kg/min to 20.5 mL/kg/min;*p* = 0.01 in empagliflozin treated patients vs. from 20.17 mL/kg/min to 19.68 mL/kg/min; *p* = 0.774 in usual care	Significant improvement in peak VO2

Data from studies on the effect of SGLT2 inhibitors on exercise performance in HF as measured with the Kansas City Cardiomyopathy Questionnaire (KCCQ); 6 min walk test (6MWT) and cardiopulmonary exercise testing (CPET) were included. Outcomes in KCCQ are displayed as OR (95% CI) unless stated otherwise; outcomes in 6MWD or peak VO2 are displayed as mean ± standard deviation or as median [IQR] unless stated otherwise. When KCCQ–clinical summary score (CSS) was not available general KCCQ-total symptom score (TSS) or KCCQ-12 score was included. Outcome of KCCQ was noted as OR (95% CI) from the latest measured timepoint of the study, unless stated otherwise. All medication dosages are depicted as daily dose in milligram (mg) unless stated otherwise. Abbreviations: sd = standard deviation; IQR = Inter quartile range; 6MWT = six minute walking test; 6MWD = six minute walking distance; CPET = Cardio Pulmonary Exercise Testing; Peak O2 = peak oxygen consumption; EOT = end of treatment; NYP = not yet published; Δ = difference from baseline; T2DM = Type 2 Diabetes Mellitus. * In these cases, HFrEF was defined as LVEF < 50%; ** Study interrupted prematurely; *** Not randomized and/or not compared to placebo.

## 5. Gaps in the Evidence and Future Perspectives

### 5.1. Summary of Effects of SGLT2 Inhibitors on Exercise Capacity

Although SGLT2 inhibitors are a topic of debate in the field of cardiology, so far little attention has been paid to their effect on exercise performance. As summarized in Table 1, exercise intolerance in HF remains difficult to treat. While many of the established cardiovascular agents have been able to improve cardiac performance and/or reduce mortality in HF, the problem of exercise intolerance remains difficult to solve. However, several of the observed effects of SGLT2 inhibitors on underlying mechanisms of exercise intolerance in HF show promising results. Although the effects on systolic function are inconsistent, a clear improvement in diastolic function has been observed after treatment with SGLT2 inhibitors. Furthermore, there is substantial evidence that cardiac remodeling can be attenuated and myocardial substrate utilization can be altered by SGLT2 inhibition. In skeletal muscle, SGLT2 inhibitors alleviate remodeling, target muscle strength, and improve muscle endurance capacity in experimental models. Moreover, SGLT2 inhibitors could improve mitochondrial function, at least in part by improving intracellular calcium handling and decreasing oxidative stress. The evident reduction in bodyweight and the re-shift towards oxidative energy production might also influence exercise intolerance favorably.

However, in the clinical setting, adequately powered studies on this topic are currently lacking. Studies with KCCQ as the outcome measure predominantly showed positive effects on the clinical symptom score. However, these studies are often substudies that were originally powered on different (cardiovascular) endpoints. Studies on the effects on 6MWT are inconclusive and do not lead to uniform results for either the effect of empagliflozin or dapagliflozin. The pilot studies that assessed exercise capacity with the ‘gold standard’ (CPET) are difficult to interpret and hypothesis-generating in nature due to the short treatment periods, the small study population, and the non-placebo-controlled study design [207,209]. The one larger RCT, however, showed a significant improvement after treatment with empagliflozin [155], suggesting that SGLT2 inhibition could be a useful tool in treating exercise intolerance in HF.

### 5.2. Future Perspectives

This review reveals divergent gaps in our knowledge of this topic of debate. First of all, the upregulation of ketone bodies has been hypothesized to be an important contributor to the cardioprotective effect of SGLT2 inhibitors [48,199]. An increase in ketone bodies has been observed in multiple studies and the cardioprotective effects of ketone bodies have also been well described. However, in the experimental setting, mechanistic studies on this topic are lacking and an animal study with a cardiac-specific BDH1 or SCOT knock-out mouse model is needed to provide robust evidence for the hypothesis that SGLT2 inhibitors work through the upregulation of ketone bodies. In the clinical setting, research on the effect of ketone body suppletion on exercise capacity in HF is still limited but would provide useful additional information on the effect of SGLT2 inhibitors on exercise performance in HF.

Furthermore, while the effects of SGLT2 inhibition on the heart have been studied in detail in the clinical population, only a few studies have focused on the effect of SGLT2 inhibitors on skeletal muscle in HF. To our knowledge, so far, no studies have examined the effect of SGLT2 inhibitors on oxidative skeletal muscle metabolism using 31P MRS testing, which would be an effective way to assess the direct effect on skeletal muscle metabolism in the clinical setting. However, as SGLT2 inhibitors have recently been included in the HF treatment guidelines for HFrEF, conducting a randomized, placebo-controlled trial in this population might be difficult. A 31P MRS study on the effect of ketone body suppletion on exercise intolerance in HF could provide additional information on the effect of ketone bodies on oxidative skeletal muscle metabolism.

Lastly, the discussed experimental study that showed that SGLT2 inhibition improves the training response suggests that SGLT2 inhibitors could potentially also play a beneficial role in the effectiveness of HF rehabilitation in the clinic [169]. This hypothesis is supported by the observed effects on the improvement of oxidative skeletal muscle metabolism and the observed switch from the glycolytic to the oxidative energy production system after treatment with SGLT2 inhibitors. Considering the effectiveness of exercise training in the HF population, it would be worthwhile to investigate whether treatment with SGLT2 inhibitors in combination with exercise therapy could further accelerate exercise tolerance in HF.

## 6. Conclusions

At present, exercise intolerance in HF remains impactful and difficult to treat. SGLT2 inhibitors are currently changing HF treatment guidelines because of their beneficial effects on cardiovascular endpoints. However, as discussed in this review, experimental and clinical findings suggest that these drugs could also ameliorate many potential targets in cardiovascular, skeletal muscle, and metabolic performance that contribute to the development of exercise intolerance. Nevertheless, little is known about the effect of SGLT2 inhibitors on exercise intolerance in HF in the clinical setting. Most of the performed studies assessed exercise tolerance using tools that do not optimally reflect exercise capacity. The limited results on the effect of SGLT2 inhibitors on cardiopulmonary exercise testing are promising and call for further research into this topic.

## Figures and Tables

**Table 1:** Overview of clinical studies on the effect of guideline-recommended heart failure medical therapy on exercise performance in HF.

Author	Medication Class	Trial	Year	Study Population	Intervention	Study Duration	Outcome CPET	Effect on Exercise
Edelmann [7]	**MRA’s**	**Aldo-DHF**	2013	422 HFpEF patients (LVEF ≥ 50%)	Spironolactone 25 mg vs. placebo	12 months	Peak VO2 changed from 16.3 ± 3.6 mL/kg/min to 16.8 ± 4.6 mL/kg/min in the spironolactone treated patients and from 16.4 ± 3.5 mL/kg/min to 16.9 ± 4.4 mL/kg/min in the placebo treated patients; *p* = 0.81.	No effect on peak VO2
Upadhya [8]			2017	71 HFpEF patients (LVEF ≥ 50%)	Spironolactone 25 mg vs. placebo	9 months	Peak VO2 was 13.5 ± 0.3 mL/kg/min in the spironolactone treated patients versus 13.9 ± 0.3 mL/kg/min in the placebo treated patients (adjusted mean difference −0.4 [−1.1 to +0.4] mL/kg/min; *p* = 0.38).	No effect on peak VO2
Kosmala [9]			2019	105 HFpEF patients (LVEF > 50%)	Spironolactone 25 mg vs. placebo	6 months	Δ peak VO2 from baseline was 3.2 ± 3.7 mL/kg/min in spironolactone treated patients vs. 0.2 ± 3.1 mL/kg/min in placebo treated patients; *p* < 0.001.	Positive effect on peak VO2
Przewlocka-Kosmala [10]			2019	114 HFpEF patients (LVEF > 50%)	Spironolactone 25 mg vs. placebo	6 months	Δ peak VO2 was 2.7 ± 3.8 mL/kg/min in spironolactone treated patients and 0.2 ± 3.1 mL/kg/min in placebo treated patients; *p* < 0.001.	Positive effect on peak VO2
Maron [11]			2018	53 HCM patients *	Spironolactone 50 mg vs. placebo	12 months	Δ peak VO2 was 0 mL/kg/min in spironolactone treated patients and +1.2 mL/kg/min in placebo treated patients; *p* = 0.7.	No effect on peak VO2
Shantsila [12]		**IMPRESS-AF RCT**	2020	250 patients with HFpEF (LVEF ≥ 55%) and permanent AF	Spironolactone 25 mg vs. placebo	2 years	Peak VO2 was 14.03 ± 5.38 mL/kg/min in spironolactone treated patients and 14.45 mL/kg/min ± 5.14 mL/kg/min in the placebo treated patients; *p* = 0.58.	No effect on peak VO2
Bruno [13] **			2018	6046 HFrEF patients (LVEF < 40%)	MRA treatment vs. no MRA treatment	Median FU: 3.8 years	Peak VO2 was 14.8 ± 4.6 mL/kg/min in MRA treated patients vs. 14.8 ± 4.7 mL/kg/min in non-MRA treated patients; *p* = 0.92.	No significant difference in VO2
Dos Santos [14]	**RAAS inhibitors**		2021	52 HFrEF patients (LVEF < 40%)	Sacubitril/valsartan 400 mg vs. enalapril 40 mg	24 weeks	Peak VO2 increased 13.5% (19.35 ± 0.99 to 21.96 ± 0.98 mL/kg/min) in sacubitril/valsartan treated patients and 12.0% (18.58 ± 1.19 to 20.82 ± 1.18 mL/kg/min) in enalapril treated patients; *p* = 0.332.	Compared to enalapril, sacubitril/valsartan did not improve peak VO2
Halle [15]		**ACTIVITY-HF**	2021	201 HFrEF patients (LVEF ≤ 40%)	Sacubitril/valsartan 400 mg vs. enalapril 20 mg	12 weeks	Δ peak VO2 was 0.55 mL/kg/min in the sacubitril/valsartan treated patients vs. 0.13 mL/kg/min in the enalapril treated patients; LS mean difference: 0.32 [−0.21 to 0.85] mL/kg/min; *p* = 0.2327.	Compared to enalapril, sacubitril/valsartan did not improve peak VO2
Butts [16] ***	**β-blockers**		2021	23 Post-operative Fontan patients	Carvedilol up to 25 mg (weight-dependent dosing) vs. placebo	12 weeks	Δ peak VO2 was −2.1 mL/kg/min in carvedilol treated patients vs. −1.42 mL/kg/min in placebo treated patients, *p* = 0.28.	No effect on peak VO2
Palau [17] ****			2022	52 HFpEF patients (LVEF > 50%)	β-blocker withdrawal vs. continuing	2 weeks	Peak VO2 was 14.3 mL/kg/min after β-blocker withdrawal and 12.2 mL/kg/min after continuation of β-blocker; Δ peak VO2 was 2.1 mL/kg/min; *p* < 0.001.	β-blocker withdrawal improved peak VO2
Dekleva [18]		**CIBIS-ELD** **substudy**	2012	30 HFrEF patients (LVEF < 45%); β-blocker naive	Bisoprolol 10 mg or carvedilol 25 mg (or 50 mg for patients > 85 kg)	12 weeks	Peak VO2 changed from 16.0 ± 3.5 mL/kg/min at baseline to 16.2 ± 3.2 mL/kg/min in the total group treated with β-blockers; *p* = 0.423.	No effect on peak VO2
Contini [19]		**The CARNEBI trial**	2013	61 HFrEF patients (LVEF ≤ 40%); for >6 mo on BB treatment	Carvedilol 25.6 mg vs. nebivolol 5.0 mg vs. bisoprolol 5.0 mg	2 months	Peak VO2 was 15.8 ± 3.6 mL/kg/min in carvedilol treated patients, 16.9 ± 4.1 mL/kg/min in nebivolol treated patients, and 16.9 ± 3.6 mL/kg/min in bisoprolol treated patients. Peak VO2 was lower in carvedilol compared to bisoprolol and nebivolol treated patients; *p* < 0.0001.	Peak VO2 was lower in carvedilol treated patients compared to Bisoprolol and Nebivolol treated patients (*p* < 0.0001)
Conraads [20]		**ELANDD**	2014	116 HFpEF patients (LVEF > 45%)	Nebivolol 5 mg vs. placebo	6 months	Peak VO2 changed from 17.02 ± 4.79 mL/kg/min to 16.32 ± 3.76 mL/kg/min in the nebivolol treated patients vs. from 17.79 ± 5.96 mL/kg/min to 18.59 ± 5.64 mL/kg/min in the placebo treated patients; *p* = 0.63.	No effect on peak VO2
Kosmala [21]	**Ivabradine**		2013	61 HFpEF patients (LVEF ≥ 50%)	Ivabradine 10 mg vs. placebo	7 days	Peak VO2 was 14.0 ± 6.1 mL/kg/min in the ivabradine treated patients vs. 17.0 ± 3.3 mL/kg/min, in the placebo treated patients; *p* = 0.001.	Short-term treatment with ivabradine increased peak VO2 compared to placebo
Pal [22]			2015	22 HFpEF patients (LVEF ≥ 50%)	Ivabradine 15 mg vs. placebo	2 weeks	Δ peak VO2 was −2.1 (−2.9 to 0) mL/kg/min in ivabradine treated patients vs. 0.9 (−0.6 to 2.1) mL/kg/min in placebo treated patients; *p* = 0.003.	Ivabradine decreased peak VO2 compared with placebo
Villacorta [23]			2018	21 HFrEF patients (LVEF < 50%)	Ivabradine 10 mg vs. pyridostigmine 90 mg	6 months	Peak VO2 changed from 13.1 mL/kg/min to 15.6 mL/kg/min; *p* = 0.048 in the ivabradine treated patients vs. 13.3 mL/kg/min to 16.7 mL/kg/min; *p* = 0.032 in the pyridostigmine treated patients.	Peak VO2 was increased in both groups
De Masi De Luca [24]			2012	111 HFpEF patients (LVEF ≥ 50%)	Ivabradine 15 mg vs. placebo	2 months	Peak VO2 changed from 16.1 ± 2.8 mL/kg/min to 19.3 ± 3.3 mL/kg/min; *p* < 0.05 in ivabradine treated patients vs. 15.7 ± 3.1 mL/kg/min to 16.0 ± 2.3 mL/kg/min; *p* = n.s. in placebo treated patients.	Peak VO2 was increased after treatment with ivabradine vs. placebo
Lewis [25]	Omecamtiv mecarbil *****	**METEORIC-HF**	2022	276 HFrEF patients (LVEF ≤ 35%)	Omecamtiv mecarbil 50–100 mg (plasma concentration dependent) vs. placebo	20 weeks	Δ peak VO2 was −0.24 mL/kg/min in omecamtiv mecarbil treated patients and 0.21 mL/kg/min in placebo treated patients; least square mean difference was −0.45 mL/kg/min [95% CI, −1.02 to 0.13]; *p* = 0.13.	No effect on peak VO2

Data from trials on the effect of guideline-recommended heart failure agents on exercise capacity in heart failure. Only randomized controlled trials, published within 10 recent years, measuring exercise by cardiopulmonary exercise testing were included, unless stated otherwise. Outcomes are displayed as mean ± standard deviation or median (IQR) unless stated otherwise. All medication dosages are depicted as daily dose in milligram (mg). Abbreviations: CPET = Cardio Pulmonary Exercise Testing; HFrEF = Heart Failure with reduced Ejection Fraction; HFpEF = Heart Failure with Preserved Ejection Fraction; HFnEF = Heart Failure with Normal Ejection Fraction; LVEF = Left Ventricular Ejection Fraction; AF = atrial fibrillation; VO2 = oxygen uptake; FU = Follow-Up; RAAS = Renin Angiotensin Aldosterone System; MRA = Mineralocorticoid Receptor Antagonist; BB = β-blocker; Δ = difference from baseline to end of treatment period. * No LVEF inclusion criterium; mean LVEF at baseline were 65% ± 3% and 64% ± 5%; ** Retrospective propensity score analysis; *** Cross-over design with washout period of 6 weeks; **** Cross-over design with washout period of 2 weeks; ***** Not yet licensed for use in heart failure.

## Data Availability

Not applicable.
